# Association between investigator-measured body-mass index and colorectal adenoma: a systematic review and meta-analysis of 168,201 subjects

**DOI:** 10.1007/s10654-017-0336-x

**Published:** 2017-12-29

**Authors:** Martin Chi-sang Wong, Chun-hei Chan, Wilson Cheung, Din-hei Fung, Miaoyin Liang, Jason Li-wen Huang, Yan-hong Wang, Johnny Yu Jiang, Chun-pong Yu, Harry Haoxiang Wang, Justin Che-yuen Wu, Francis Ka-leung Chan, Joseph Jao-yiu Sung

**Affiliations:** 1 Institute of Digestive Disease, Faculty of Medicine, Chinese University of Hong Kong, Shatin, China; 2 School of Public Health and Primary Care, Faculty of Medicine, Chinese University of Hong Kong, Shatin, China; 3 School of Basic Medicine, Peking Union Medical College and Institute of Basic Medical Sciences, Chinese Academy of Medical Sciences, Beijing, 100050 China; 4 Peking Union School of Public Health, Chinese Academy of Medical Sciences and Peking Union Medical College, Beijing, 100050 China; 5 Li Ping Medical Library, Chinese University of Hong Kong, Shatin, HKSAR China; 6 School of Public Health, Sun Yat-sen University, Guangzhou, 510080 Guangdong China; 7 Department of Medicine and Therapeutics, Faculty of Medicine, Chinese University of Hong Kong, Shatin, China; 8 State Key Laboratory of Digestive Disease, Faculty of Medicine, Chinese University of Hong Kong, Shatin, China

**Keywords:** Body mass index, Colorectal adenoma, Association, Meta-analysis

## Abstract

The objective of this meta-analysis is to evaluate the odds of colorectal adenoma (CRA) in colorectal cancer screening participants with different body mass index (BMI) levels, and examine if this association was different according to gender and ethnicity. The EMBASE and MEDLINE were searched to enroll high quality observational studies that examined the association between investigator-measured BMI and colonoscopy-diagnosed CRA. Data were independently extracted by two reviewers. A random-effects meta-analysis was conducted to estimate the summary odds ratio (SOR) for the association between BMI and CRA. The Cochran’s Q statistic and I^2^ analyses were used to assess the heterogeneity. A total of 17 studies (168,201 subjects) were included. When compared with subjects having BMI < 25, individuals with BMI 25–30 had significantly higher risk of CRA (SOR 1.44, 95% CI 1.30–1.61; I^2^ = 43.0%). Subjects with BMI ≥ 30 had similarly higher risk of CRA (SOR 1.42, 95% CI 1.24–1.63; I^2^ = 18.5%). The heterogeneity was mild to moderate among studies. The associations were significantly higher than estimates by previous meta-analyses. There was no publication bias detected (Egger’s regression test, *p* = 0.584). Subgroup analysis showed that the magnitude of association was significantly higher in female than male subjects (SOR 1.43, 95% CI 1.30–1.58 vs. SOR 1.16, 95% CI 1.07–1.24; different among different ethnic groups (SOR 1.72, 1.44 and 0.88 in White, Asians and Africans, respectively) being insignificant in Africans; and no difference exists among different study designs. In summary, the risk conferred by BMI for CRA was significantly higher than that reported previously. These findings bear implications in CRA risk estimation.

**Electronic supplementary material:**

The online version of this article (10.1007/s10654-017-0336-x) contains supplementary material, which is available to authorized users.

## Introduction

Colorectal cancer (CRC) is a leading cause of morbidity and mortality on a global scale [1]. Its incidence is rising rapidly in many low- and middle-income countries [2], as well as Asia Pacific nations such as Japan, Korea, Singapore and Hong Kong [3, 4]. Overweight and obesity, defined as a body mass index (BMI) of 25–30 and ≥ 30 kg/m^2^, respectively, is one of the recognized environmental risk factors for the development of CRC [5–7]. Whilst obesity is preventable, statistics from the World Health Organization reported that more than 1.9 billion adults aged 18 years or older (39%) were overweight in 2014; amongst them over 600 million (13%) were obese [8]. Its increasing prevalence has been regarded as a major contributor to the rising trend of CRC.

Colorectal adenomas (CRA) are present in more than 30% of asymptomatic general populations [9]. Among all CRC screening participants who received colonoscopy with polyps detected, CRA is amongst the most frequent pathological findings [10]. Since most CRCs develop via genetic and morphological adenoma-carcinoma progression from CRAs, it is widely accepted that both CRCs and CRAs share similar epidemiological features and etiological causes. Hence, some risk algorithms have adopted BMI as a predictor variable to risk-stratify subjects for colorectal neoplasia [10].

Nevertheless, the association between BMI and CRA has not been consistently demonstrated in all populations [11–28]. Some studies reported a significant association between BMI and CRA [11, 12, 14–21, 23–26, 28] whilst others did not [13, 22, 27, 29–31]. Two recent meta-analyses have been performed to pool data from published studies on the relationship between BMI and CRA. In 2012, Okabayashi and colleagues systematically reviewed 23 studies (105,190 participants) in their meta-analysis on the prediction value of BMI for CRA, and revealed a dose–response relationship where the risk of CRA increased with higher BMI levels [32]. However, there exist major limitations as self-reported BMI was used, and this could lead to misclassification of BMI categories in public health research [33]. In that meta-analysis [32], a significant proportion of studies included used self-reported questionnaires to determine BMI and the presence of CRA, and this could reduce the robustness of the conclusions drawn due to possible reporting bias. In another systematic review with the same research objective, the limitation of relying on questionnaire surveys to measure BMI or CRA was noticed in 15 of 36 included studies [34]. Since the publication of these two meta-analyses, there are 10 additional studies that were published including large number of screening participants using physician-measured BMI and colonoscopy diagnosed CRA as inclusion criteria. For instance, a multi-centre study in 16 Asia Pacific countries recruited more than 11,797 asymptomatic screening participants who received colonoscopies, and the study was published in 2016 [35]. The precise magnitude of the association between BMI and CRA remains unknown, and whether there exist differences in this association in subjects with different characteristics is yet to be explored. This knowledge gap is important as it bears clinical implications in formulation of risk scores for CRA in different patient groups, and informs clinical guidelines regarding target groups for priority screening. This meta-analysis aims to evaluate the odds of colorectal adenoma (CRA) in colorectal cancer screening participants with different body mass index (BMI) levels, and examine if this association was different according to gender and ethnicity.

## Methods

### Literature search strategy

We conducted the literature search by systematically searching MEDLINE (from 1946 to March 2017), EMBASE (from 1974 to March 2017) and by hand searching the reference lists of original studies and review articles on this topic. Our search terms consisted of three main components, colorectal (colorectal OR colon OR colonic OR rectum OR rectal) AND disease (cancer* OR neoplas* OR tumor* OR tumour* OR carcinoma* OR sarcoma* OR adenoma* OR lesion* OR polyp* OR CRC) AND obesity or overweight (body mass index OR BMI OR body size OR body weight OR intraabdominal OR overweight OR fat OR obesity OR obese OR waist) [32] (Supplementary File 1). Grey literature search was performed in Grey Literature Report (www.greylit.org), related thesis, and conference reports. No language restrictions were imposed.

### Inclusion and exclusion criteria

CRA, defined as the presence of either non-advanced or advanced adenoma, is the primary outcome of this study. We included all cross-sectional studies, case control studies and cohort studies that examined the relationship between BMI and the prevalence of CRA. In these studies, odds ratios (OR) with 95% confidence intervals (CI) between CRA and BMI categories were recorded. We excluded the following studies: (1). those with hyperplastic polyps, serrated adenomas or CRC cases as majority of all lesions; (2). those where subjects had higher CRC risk as compared to the general population, for instance, individuals with a family history of CRC in first-degree relatives; (3). those that could not generate OR for BMI category and CRAs; (4). those with symptomatic participants; (5). those with BMI data obtained from self-reported questionnaires; (6). those with CRA not diagnosed by colonoscopy and histological examination; (7). those with CRA data not derived from the whole colon and rectum. The eligibility of studies was assessed by two investigators (J. L. H. and C. H. C.) and in cases of disagreement, consensus was made via referral to a third reviewer (M. C. S. W.). We attempted to contact authors of studies if there were any missing data.

### Quality assessment of selected studies

The Newcastle Ottawa Scale (NOS) was employed to evaluate the quality of the included studies according to their design by two assessors (J. L. H. and C. H. C.) who are librarian experts [36, 37]. The NOS was used to confirm that the included studies are of high quality, which was scored based on the summation of items described below. Similar items among different study types for quality assessment were as follows: (1) representativeness of the samples: one point was assigned if the subjects represent the general population/case group/controls group/exposed cohort/non-exposed cohort. No points were assigned if samples are special population groups (e.g. veteran) or not mentioned; (2) ascertainment of the exposure: one point was assigned if measurement of BMI was performed by healthcare professionals, 0 point was assigned if BMI was self-reported or not specified. Since all our included studies measured BMI by healthcare professionals, none was assigned 0 point; (3) comparability: for subjects in different outcome groups or case/control groups, two points were assigned for adequate adjustment of recognized risk factors for colorectal adenoma; one point for adjustment of some covariates only, and zero point for no adjustment; (4) assessment of the outcome: colonoscopy and histological examination: one point was assigned if it was based on medical records or histology report, no point was given if the assessment was self-reported by study participants or not specified.

For cross-sectional studies, additional items for quality assessment include: (1) sample size: if the sample size is justified, one point was assigned, otherwise no point was given; (2) non-respondents: one point was assigned if the response rate is satisfactory, otherwise no point; (3) assessment of the outcome: for those studies in which outcome assessment was independent and blinded, one extra point was added accordingly; (4) statistical test: one point was assigned if the statistical test is appropriate, clearly described and complete; otherwise no point was assigned. For case-control studies, additional items include: (1) same method of ascertainment for cases and controls: one point was assigned; (2) non-response rate: one point was given if the rate for both case and control groups was the same, and no point was assigned for non-respondents. For cohort studies, the additional items are: (1) demonstration that outcome of interest was not present at study commencement: one point was assigned for stating exclusion of CRA/ advanced CRA/ CRC subjects or stating subjects have no history of CRA/ advanced CRA/ CRC; (2) follow-up duration: one point was assigned for all eligible studies if the follow-up period is long enough to detect CRA; (3) adequacy of following up of cohorts: one point was assigned for completing at least 90% of follow-up. Scores ranged from 0 (lowest) to 9 (highest). Similar to previous literature [32], studies with scores ≥ 7 were classified as “high” quality and those with scores < 7 were classified as “low” quality.

### Data extraction

The characteristics of studies were recorded, including the names of the first authors, publication year, country, design, enrolment year, BMI category, strategies to capture BMI data, the definition of non-cases and the definition of advanced adenoma. BMI is categorized according to WHO classification: normal (< 25 kg/m^2^), overweight (25–30 kg/m^2^) and obese (≥ 30 kg/m^2^). The number of cases and non-cases in the 3 categories, as well as the study design, gender and subject ethnicity, were recorded if available. Data extraction and data checking were performed by 3 investigators (J. L. H., C. H. C. and W. C.)

### Statistical analysis

Random effects model meta-analysis was conducted to synthesize a summary estimate of the association between different BMI groups and CRA. Summary odds ratios (SOR) with 95% confidence intervals (CI) were used as a proxy measure for effect size, and were calculated by comparing 3 BMI categories (≥ 25, 25–30, ≥ 30 kg/m^2^) with BMI < 25 kg/m^2^. The SOR was computed with the assumption that the outcomes categorized by different BMI groups were derived from patients independently, so there was no within-study correlation of adenoma prevalence. Z-tests were used to investigate the significance of pooled estimate, and Cochran’s Q and I^2^ statistics were used to examine the heterogeneity within groups and between groups [38]. For publication bias, funnel plot asymmetry was assessed by the Egger’s and Begg’s regression test [39, 40]. Subgroup analysis was applied in this study to perform comparisons according to subsets of studies, such as study design, gender, ethnicity, types of adenoma and degree of CRA progression. We also conducted a meta-regression analysis to explore heterogeneity between the studies.

In the present study, R ver. 3.3.1 (The R Foundation for Statistical Computing) with metafor package ver. 1.9–9 was used to conduct the statistical analysis [41]. All functions were performed under restricted maximum likelihood estimation. Two-tailed *p* value < 0.05 was defined as statistical significant for all the comparisons. Heterogeneity was considered as low, moderate and high, when I^2^ was 25, 50 and 75% respectively. This systematic review was written following the PRISMA guideline [42].

## Results

### Search results and study characteristics

**Fig. 1:**
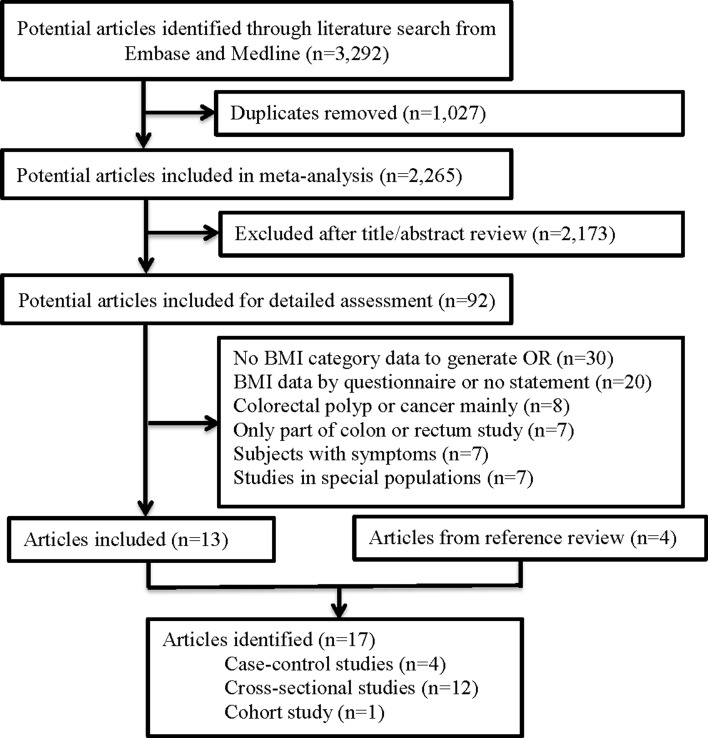
Flow diagram of study selection

The search strategy yielded 3292 citations. We removed 1027 duplicates, and 2173 articles were removed after title and abstract review (Fig. 1). A total of 92 studies were reviewed in full text and 13 studies fulfilled our eligibility criteria. Four additional articles were retrieved from review of the reference sections of original articles and grey literature search, resulting in 17 articles included for data analysis (168,201 subjects). Among them, 12 were cross-sectional studies [24, 34, 43–52], 4 were case-control studies [53–56], and one was a cohort study [57] (Tables 1, 2). The quality of all included studies was assessed by the Newcastle Ottawa Scale (NOS) (Table 3). All studies were found to have good quality, with 15 studies scoring 8 points and 2 studies scoring 7 points. Of all the 17 studies, 11 included data of Asian subjects [24, 44–51, 53, 56], 4 included data of white subjects and 4 included data of individuals of African descent [46, 52, 55, 56]. The proportion of screening participants with BMI > 25 kg/m^2^ was 29.3, 49.7 and 58.1% in Asian, African and white subjects, respectively. No studies were found to use identical cohorts. The search did not identify any studies published in grey literature.

**Table 1:** Characteristics of included studies

Authors	Year	Country	Design	Sample size	BMI category	Prevalence (%) of subjects with BMI > 25 kg/m^2^	NOS score
Guilera et al. [43]	2005	USA	1	720	18.5–24.9, 25–29.9, > 30	61.9	8
Kim et al. [44]	2007	South Korea	1	1744	18.5–23.0, 23.0–24.9, ≥ 25	31.1	8
Sedjo et al [57]	2007	USA	3	600	< 25, 25–29, 30+	82.8	8
Kim et al. [45]	2010	South Korea	1	1316	< 25, ≥ 25	30.9	8
Nam et al. [24]	2010	South Korea	1	3933	< 20, 20–24.9, 25.0–29.9, ≥ 30	33.4	8
Stein et al. [46]	2010	USA	1	600	< 25, 25–30, 30–35, ≥ 35	68.3	8
Kim et al. [47]	2011	South Korea	1	1322	< 23, 23.0–24.9, ≥ 25	37.4	8
Kim et al. [48]	2012	South Korea	1	3430	18.5–25, ≥ 25	29.9	8
Choe et al. [53]	2013	South Korea	2	1206	≤ 22.9, 23.0–24.9, 25.0–29.9, ≥ 30	34.4	8
Czwornog et al. [54]	2013	USA	2	773	18.5–25, 25.0–30, ≥ 30	72.7	8
Lipka et al. [55]	2013	USA	2	779	<18.5, 18.5–24.9, 25.0–29.9, > 30.0	82.4	7
Yun et al. [49]	2013	South Korea	1	18,085	< 18.5, 18.5–22.9, 23.0–24.9, ≥ 25	18.4	8
Lee et al. [50]	2014	South Korea	1	1574	< 23, 23–25, ≥ 25	32.0	8
Wang et al. [51]	2014	Taiwan	1	1894	< 25, 25–30, > 30	34.1	8
Murphy et al. [52]	2015	South Korea	2	3561	< 25, ≥ 25	60.2	8
Kim et al. [56]	2015	USA	1	2184	18.5–25, 25–30, > 30	33.4	7
Wong et al. [35]	2016	Asia Pacific	1	11,362	< 25, 25–30, ≥ 30	32.2	8

Design: 1 cross-sectional, 2 case-control, 3 Cohort

*BMI* body mass index, *NOS scale* the Newcastle–Ottawa scale

**Table 2:** Pathology findings from included studies

Author	Polyp-free (n, %)	HP (n, %)	Non-AA (n, %)	AA (n, %)	CRC (n, %)	Definition of normal	Definition of AN
Guilera et al. [43]	494 (68.6)^#^		226 (31.4)	NS	NS	1	NS
Kim et al. [44]	1460 (83.7)	NS	206 (11.8)	78 (4.5)	NS	2	AA, CRC
Sedjo et al. [57]	410 (68.3)	54 (9.0)	98 (16.3)	38 (6.3)	0	1	AA
Kim et al. [45]	1053 (80.0)	Excluded	228 (17.3)	35 (2.7)	Excluded	2	AA
Nam et al. [24]	2877 (73.2)	NS	960 (24.4)	85 (2.2)	11 (0.3)	2	AA, CRC
Stein et al. [46]	384 (64.0)	NS	176 (29.3)	40 (6.7)	0	2	AA, CRC
Kim et al. [47]	908 (68.7)	Excluded	368 (27.8)	46 (3.5)	Excluded	2	AA
Kim et al. [48]	2456 (71.6)^#^		744 (21.7)	224 (6.5)	6 (0.2)	2	AA, CRC
Choe et al. [53]	557 (46.2)	NS	554 (45.9)	NS	153 (12.7)	3	NS
Czwornog et al. [54]	567 (73.4)	NS	206 (26.6)	NS	NS	1	AA (any size)
Lipka et al. [55]	612 (78.6)	NS	167 (21.4)	NS	Excluded	1	NS
Yun et al. [49]	16,163 (89.4)^#^		1674 (9.3)	248 (1.4)	Excluded	3	AA, CRA ≥ 3
Lee et al. [50]	1080 (68.6)^#^		494 (31.4)	NS	Excluded	1	NS
Wang et al. [51]	1379 (72.8)	210 (11.1)	305 (16.1)*		NS	3	NS
Murphy et al. [52]	3129 (87.9)	NS	685 (19.2)	143 (4.0)	13 (0.4)	2	AA, CRC
Kim et al. [56]	1555 (71.2)	NS	629 (28.8)	NS	NS	1	NS
Wong et al. [35]	7177 (63.2)	853 (7.5)	2604 (22.9)	657 (5.8)	71 (0.6)	2	AA

Normal definition: 1: non-adenomatous; 2: polyps-free; 3: normal findings

*HP* hyperplastic polyp, *AN* advanced neoplasia, *CRA* colorectal adenoma, *CRC* colorectal cancer, *AA* advanced adenoma, adenoma measuring > 10 mm in diameter and/or with villous components and/or showing high grade dysplasia (32)

^#^Mixed with polys-free and HP (hyperplastic polyp)

*Mixed with any adenomas

**Table 3:** Quality assessment of included studies based on the Newcastle Ottawa Scale (NOS)

	Selection (4)				Comparability (2)	Exposure (3)			Total score
	Is the case definition adequate (assessment of outcome)	Representativeness of the cases	Selection of controls (assessment of outcome)	Definition of controls (representativeness of the samples)	Adjusted for covariates	Ascertainment of exposure	Same method of ascertainment for cases and controls	Non-response rate	
*Case*-*control studies*									
Czwornog et al. [54]	1	1	1	1	2	1	1	0	8
Choe et al. [53]	1	1	1	1	2	1	1	0	8
Lipka et al. [55]	1	1	0	1	2	1	1	0	7
Kim et al. [56]	1	1	1	1	2	1	1	0	8

	Selection (4)				Comparability (2)	Outcome (3)		Total score
	Representativeness of the samples	Sample size	Non-respondents	Ascertainment of the exposure	Adjusted for covariates	Assessment of the outcome (2)	Statistical test	
*Cross*-*sectional studies*								
Guilera et al. [43]	1	1	0	1	2	2	1	8
Kim et al. [44]	1	1	0	1	2	2	1	8
Kim et al. [45]	1	1	0	1	2	2	1	8
Nam et al. [24]	1	1	0	1	2	2	1	8
Stein et al. [46]	1	1	0	1	2	2	1	8
Kim et al. [47]	1	1	0	1	2	2	1	8
Kim et al. [48]	1	1	0	1	2	2	1	8
Yun et al. [49]	1	1	0	1	2	2	1	8
Lee et al. [50]	1	1	0	1	2	2	1	8
Wang et al. [51]	1	1	0	1	2	2	1	8
Murphy et al. [52]	0	1	0	1	2	2	1	7
Wong et al. [59]	1	1	0	1	2	2	1	8

	Selection (4)				Comparability (2)	Outcome (3)			Total score
	Representativeness of the exposed cohort	Selection of the non-ex-posed cohort	Ascertainment of exposure	Demonstration that outcome was not present at start of study	Adjusted for covariates	Assessment of outcome	Follow-up long enough for outcomes to occur	Adequacy of follow up of cohorts	
*Cohort studies*									
Sedjo et al [57]	1	1	1	1	2	1	1	0	8

### The association between body mass index and colorectal adenoma

**Fig. 2:**
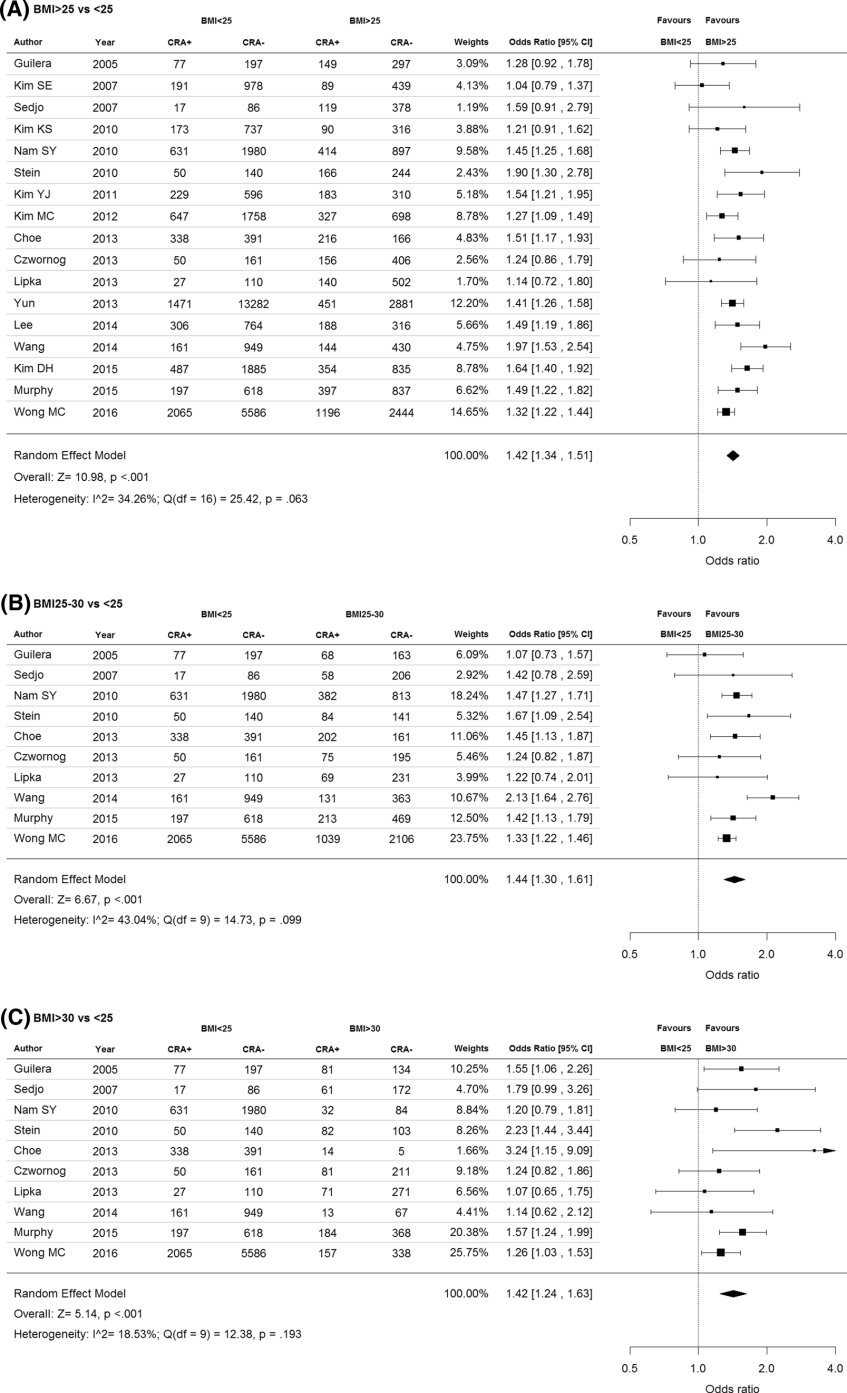
Odds ratios for colorectal adenoma (**a** BMI ≥ 25 vs. <25; **b** BMI 25–30 vs. < 25; **c** ≥ 30 vs. < 25)

Meta-analysis of the included articles via a random-effects model showed a SOR of 1.42 (95% CI 1.34, 1.51) among subjects with BMI ≥ 25 compared to subjects with BMI < 25, where the heterogeneity was moderate and statistically insignificant (I^2^ = 34.3%, p_heterogeneity_ = 0.063) (Fig. 2a). Using BMI < 25 as a reference, the associations with any CRA were similar between those with BMI 25–30 (SOR 1.44, 95% CI 1.30, 1.61; I^2^ = 43.0%, p_heterogeneity_ = 0.099; Fig. 2b) and BMI ≥ 30 (SOR 1.42, 95% CI 1.24, 1.63; I^2^ = 18.5%, p_heterogeneity_ = 0.193; Fig. 2c). No statistically significant difference were found between the two groups [*p* difference = 0.887]. All 17 studies reported data on CRA among subjects with BMI > 25, but only 10 studies reported number of CRA among subjects with BMI 25–30 and BMI > 30 kg^2^/m. The magnitude of association was similar between different BMI groups and non-advanced adenoma (BMI ≥ 25 vs. < 25: SOR 1.36, 95% CI 1.26, 1.47; BMI 25–30 vs. < 25: SOR 1.33, 95% CI 1.22, 1.47; BMI ≥ 30 vs. < 25: SOR 1.38, 95% CI 1.04, 1.84) and did not show statistically significant difference when compared with any CRA. When compared with subjects with BMI < 25, the odds of advanced adenoma was significantly higher among those with BMI ≥ 25 (SOR 1.52, 95% CI 1.32, 1.73). The relationship between BMI and advanced adenoma using “non-advanced adenoma” as non-cases did not show statistical significance.

### Subgroup analysis

**Fig. 3:**
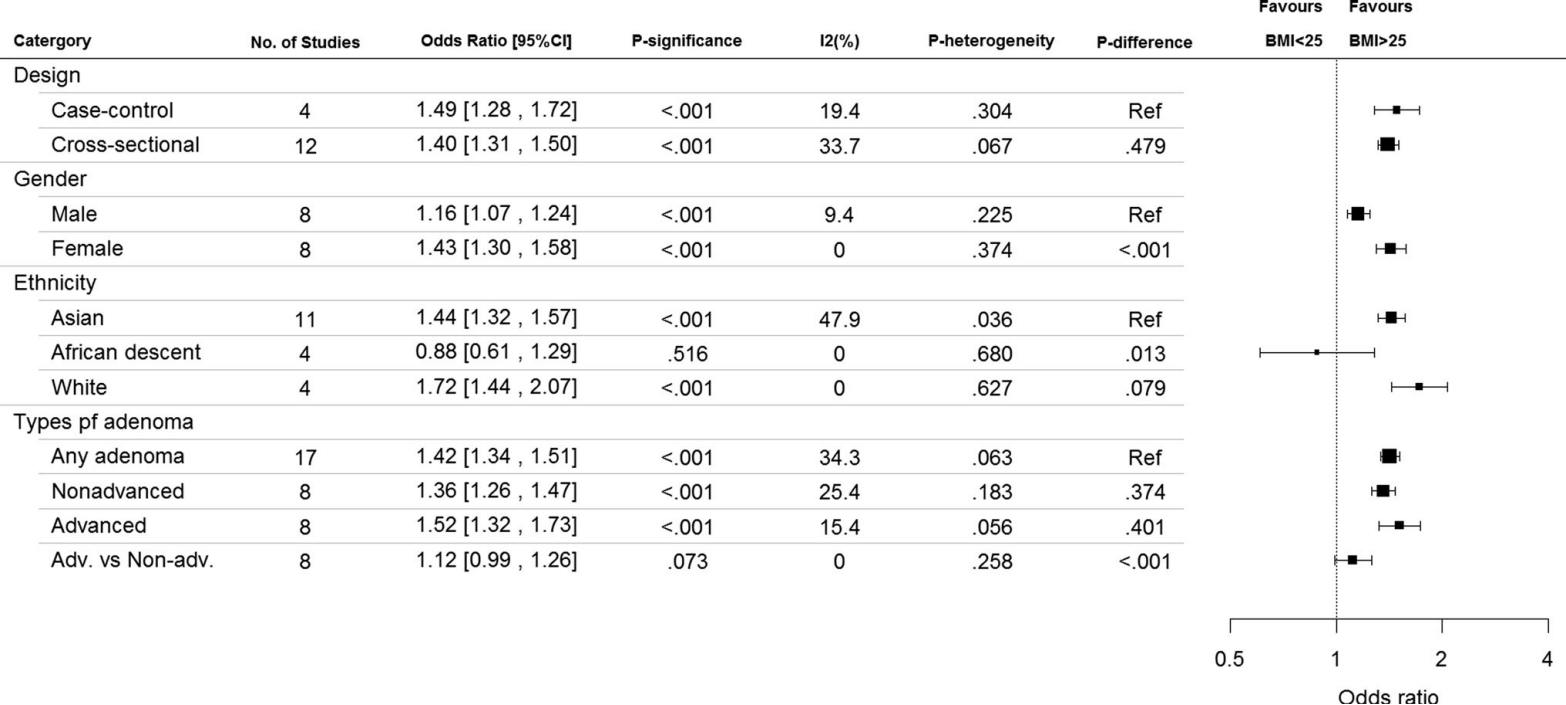
Subgroup analysis – association between BMI and colorectal adenoma according to study design, gender, ethnicity, and types of adenoma (BMI ≥ 25 vs. < 25)

Eight studies examined the association in men and women separately, and it was found that female subjects had significantly higher odds of CRA (SOR 1.43, 95% CI 1.30, 1.58) when compared with men (SOR 1.16, 95% CI 1.07, 1.24; between-groups *p* difference of < 0.001) (Fig. 3). Among subjects of white ethnicity (SOR 1.72, 95% CI 1.44, 2.07) and Asian ethnicity (SOR 1.44, 95% CI 1.32, 1.57), individuals with BMI > 25 kg/m^2^ had higher odds of CRA than those with BMI < 25 kg/m^2^. The odds was higher compared to Africans but the findings indicated only a significant difference between Asian and Africans. The SORs between BMI and CRA showed no statistically significant difference between cross-sectional and case control studies (*p* = 0.479). Meta-regression analysis based on BMI 25–30 as a reference and BMI>30 kg/m^2^ implied that different levels of BMI could not explain the heterogeneity observed in this meta-analysis (coefficient − 0.01 [95% CI − 0.20, 0.18], *p* = 0.905).

### Publication bias

**Fig. 4:**
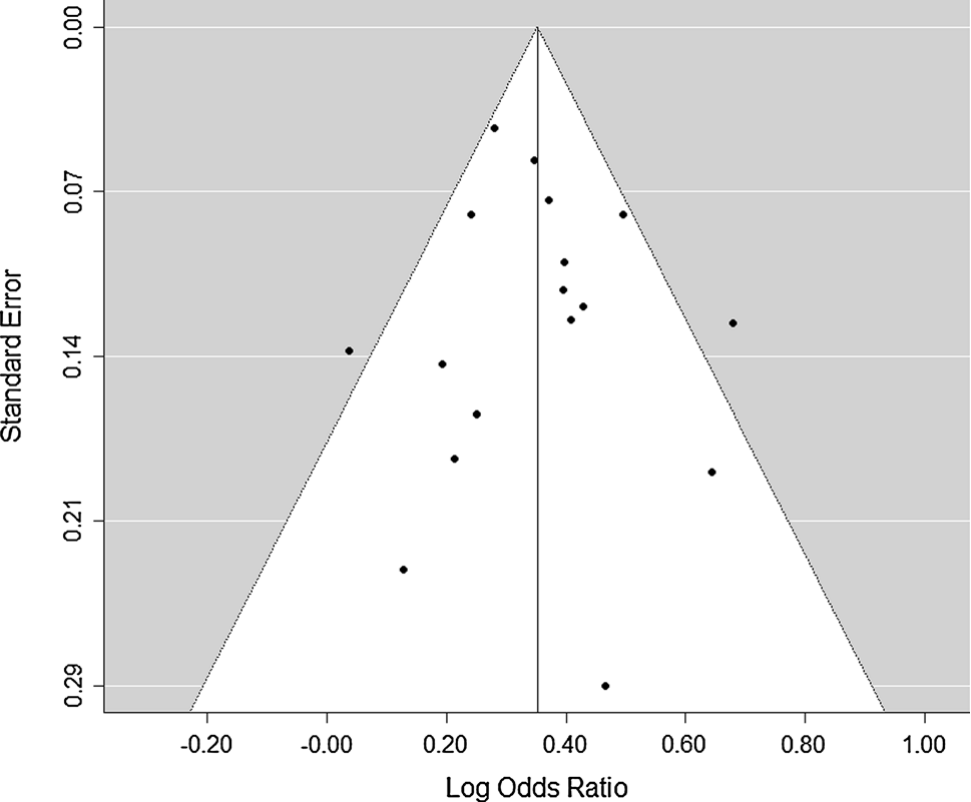
Funnel Plot for identification of publication bias

The Egger’s test (t = − 0.560, *p* = 0.584) and Begg’s test (Kendall’s tau = 0.059, *p* = 0.777) for funnel plot asymmetry identified insignificant publication bias (Fig. 4). There were two outliers in the funnel plot, and the trim and fill analysis showed no missing studies. When these two outliers [44, 51] were excluded and the association between any CRA and BMI (≥ 25 vs. < 25) was re-examined, the SOR was 1.41 (95% CI 1.34, 1.49) which was statistically similar to the SOR computed from all studies.

## Discussion

This systematic review and meta-analysis based on high quality studies reported increased risks of any CRA and non-advanced adenomas in the overweight and obese populations by a magnitude of 33–44%—risk estimates that are significantly higher than those reported previously. BMI was found to be a significant factor associated with detection of CRA in terms of its magnitude, and hence should be considered as an important factor in risk algorithms predicting the risk of CRA. The strength of association between BMI and CRA was higher in female subjects and individuals of western or Asian ethnicities, but was insignificant in subjects of African descent.

This meta-analysis is distinct from previous systematic reviews by restricting analysis to the most updated studies retrieved from a broad search strategy that included the most comprehensive data. This enables more robust evaluations on the association between BMI and CRA, allowing a more precise magnitude to be determined. Several limitations should, nevertheless, be addressed. Firstly, the assessment of BMI and CRA might not be universally standardized among different studies, and it is well recognized that there is a higher likelihood for obese patients, or subjects with different characteristics, to present with poorer bowel preparation at colonoscopy procedures [58, 59]. Therefore, the summary odds ratios identified in the present study might have been underestimated. Second, the calendar years where CRA were detected are different across studies, where colonoscopists with different levels of experience and expertise were involved. The adenoma detection rate might increase with time due to higher prevalence with rapid urbanization and more affluent lifestyles. Also, there have been very few prospective cohort studies that followed-up screening subjects and examine the direct influence of obesity on CRA development [57]. Furthermore, the estimation of dose-response association requires at least three non-reference dose levels [60]. As most original studies included in this meta-analysis only used two non-reference dose levels (BMI 25–30, BMI > 30, reference: BMI < 25), dose-response meta-analysis could not be performed. From one cohort study (Sedjo et al. [57]), the association between CRA and obesity vs. overweight (adjusted OR 2.16, 95% CI 1.13–4.14 vs. OR 1.54, 95% CI 0.81–2.91) suggested a trend towards dose-response relationship, although statistical analysis did not confirm such relationship. The cross-sectional nature of most studies included in this meta-analysis might obscure a potential dose-response association. In addition, multivariate meta-regression analysis could not be performed since we need an appropriately large ratio of studies to covariates [61]. In this meta-analysis it is not feasible due to multiple covariates and the small number of studies. Lastly, as the majority of studies included in this meta-analysis are cross-sectional or case-control studies, one could not infer a cause-and-effect relationship between BMI and CRA.

The exact mechanisms of colorectal carcinogenesis induced by obesity are still not entirely clear. Our study findings reported a significant association between BMI and CRA, but when the outcome measure is development of non-advanced CRA to advanced CRA, the association becomes insignificant. This implies that obesity could exert, to a larger extent, its influence on risk of adenoma, but less so on adenoma progression. There has been a postulation that genetic alteration like the common single-nucleotide polymorphism variants around the melanocortin 4 receptor gene could be associated with the co-occurrence of obesity and CRA [32, 62]. Alternatively, it has been hypothesized that insulin resistance and subsequent hyperinsulinemia induced by obesity may lead to direct mitogenic and antiapoptotic signaling by insulin or insulin-like growth factor axis [63, 64]. Furthermore, obesity has been regarded as a condition of chronic low-grade inflammation with elevation of pro-inflammatory cytokines, including tumor necrosis factor and interleukin-6. These inflammatory mediators have direct tumorigenic effects on the gastrointestinal tract [63, 64]. From a recent meta-analysis, leptin and adiponectin have also been implicated in the pathogenesis of CRA in obese patients [65]. In addition, there are metabolic, lipidomic and transcriptomic differences between visceral adipose tissue (VAT) and subcutaneous adipose tissue (SAT) compartments in colorectal carcinogenesis [66], which have not been differentiated in this study. There is emerging evidence demonstrating that the relationship between obesity and cancer is mediated by VAT rather than SAT. Several studies have identified a unique role of VAT in the risk and progression of CRC. It has been postulated that VAT alters metabolic activity and induces chronic systemic inflammation that promotes a pro-oncogenic environment [67]. Future studies may explore the magnitude of association between VAT and CRA.

We found that a 5-unit increase of BMI conferred an up to 44% increased risk for CRA. This additional risk is significantly higher than that estimated by previous meta-analyses [9, 32]. The increased risk estimated by Okabayashi et al and Ben et al in 2012 was 24 and 19%, respectively. The difference could be explained by different inclusion criteria of original studies in these meta-analyses. In their evaluations, studies that included self-reported BMI and questionnaire-measured CRA were also included in their systematic review. Studies showed that BMI based on self-reports were more frequently under-reported, where data from measurement devices usually revealed higher proportions of overweight and obesity [68, 69]. Hence, the true association between BMI and CRA might be biased towards lower risk. In addition, except on cohort study, this meta-analysis mainly included case-control and cross-sectional studies. Risk estimates are therefore higher in retrospective studies as compared to previous meta-analyses, which also included prospective studies.

Our study also found that the association between BMI and CRA was significantly higher in women than men, in the context of higher prevalence of CRC in men when compared with women. It has been suggested that this gender difference might be due to the role of endogenous and exogenous sex hormones on the adenocarcinoma sequence [32]. It is well recognized that pre-menopausal women had a stronger susceptibility to CRA development due to endogenous estrogen secretion, where activation of estrogen receptor-α leads to increase in gene transcription and cancer proliferation [70]. As for the differences in the association between BMI and CRA, ethnicity of individuals was found to be a significant effect modifier. In particular, the association between BMI and CRA was found to be absent in subjects of African descent. The difference in prevalence of overweight and obesity in individuals according to ethnicity might affect the comparability among studies that included screening participants of different ethnic groups. From existing literature, the magnitude of this association has not been adequately examined, and the exact reasons of this observation will need to be explored in future studies.

These study findings showed that being overweight (BMI 25–30) is associated with similar risk for CRA when compared with obesity (BMI ≥ 30), and hence bring forth an alert to physicians and public health practitioners on early intervention for overweight patients in order to reduce future risk of CRA. In addition, our data showed that risk algorithms for CRA would need to take gender and ethnicities into account for more accurate risk prediction, and these findings could be used for devising such risk-stratification scores. Future studies should examine the mechanistic aspects of the differential effects of these variables on CRA development. As there is a scarcity of prospective studies on the impact of BMI on progression of CRA to advanced CRA, additional longitudinal cohort evaluations should be performed with strategies that address confounding and selection biases.

## Electronic supplementary material

Below is the link to the electronic supplementary material.

Supplementary material 1 (DOCX 16 kb)

Supplementary material 2 (DOCX 40 kb)

## Abbreviations

BMI: Body-mass index
CRA: Colorectal adenoma
NOS: Newcastle Ottawa Scale (NOS)
SOR: Summary odds ratio
SAT: Subcutaneous adipose tissue
VAT: Visceral adipose tissue
