# Supplementary material for: Association between investigator-measured body-mass index and colorectal adenoma: a systematic review and meta-analysis of 168,201 subjects
Source: Eur J Epidemiol. 2017 Dec 29;33(1):15–26. doi: 10.1007/s10654-017-0336-x (PMC5803281; doi:10.1007/s10654-017-0336-x)
Supplement: Supplementary file 1 — Supplementary material 1 (DOCX 16 kb) [file 10654_2017_336_MOESM1_ESM.docx]

**Supplementary File 1 Search Strategy**

Database: Embase (1910 to Present), MEDLINE (1946 to Present with Daily Update)

Date: from inception to Nov 27, 2016

1. (body mass index or BMI or body size or body weight or intraabdominal or overweight or fat or obesity or obese or waist).mp.
2. (colorectal or colon or colonic or rectum or rectal).mp.
3. (cancer* or neoplas* or tumor* or tumour* or carcinoma* or sarcoma* or adenom* or lesion* or polyp* or CRC).mp.
4. ((cohort adj (study or studies)) or "case control" or "cohort analy*" or (observational adj (study or studies)) or longitudinal or retrospective or "cross sectional" or cross-sectional or (follow up adj (study or studies))).mp.
5. 2 and 3
6. 1 and 4 and 5
7. limit 6 to full text
8. limit 7 to human
