# Supplementary material for: Association between investigator-measured body-mass index and colorectal adenoma: a systematic review and meta-analysis of 168,201 subjects
Source: Eur J Epidemiol. 2017 Dec 29;33(1):15–26. doi: 10.1007/s10654-017-0336-x (PMC5803281; doi:10.1007/s10654-017-0336-x)
Supplement: Supplementary file 2 — Supplementary material 2 (DOCX 40 kb) [file 10654_2017_336_MOESM2_ESM.docx]

### **Supplementary File 2: Studies excluded from the meta-analysis according to exclusion criteria**

### **(1). those with hyperplastic polyps, serrated adenomas or colorectal cancer cases as majority of all lesions (n=8):**

1. Chung, Yong Woo, et al. Association of obesity, serum glucose and lipids with the risk of advanced colorectal adenoma and cancer: a case-control study in Korea. *Digestive and Liver Disease* 2006;**38**:668-672.
2. Ji J H, Park B J, Park Y S, et al. Clinicopathologic study of colorectal polyps and obesity in Korean adult. *The Korean Journal of Gastroenterology* 2007;**49**:10-16.
3. Kim, Ji Yeon, et al. Different risk factors for advanced colorectal neoplasm in young adults. *World Journal of Gastroenterology* 2016;**22**:3611-3620.
4. Park Y, Lee J, Oh J H, et al. Dietary patterns and colorectal cancer risk in a Korean population: a case-control study. *Medicine* 2016;**95**:e3759
5. Teetzmann A, Koch H, Oehler G. Coherence between body composition and benign colorectal neoplasia. *Med Welt* 2006;**57**:281–283.
6. Wang, Fu-Wei, et al. Prevalence and risk factors of asymptomatic colorectal polyps in Taiwan. *Gastroenterology Research and Practice* 2014;**15**:e5205
7. Wise, Lauren A., et al. Anthropometric risk factors for colorectal polyps in African‐American women. *Obesity* 2008;**16**:859-868.
8. Wong M C S, Lam T Y T, Tsoi K K F, et al. Predictors of advanced colorectal neoplasia for colorectal cancer screening. *American Journal of Preventive Medicine* 2014;**46**:433-439.

### **(2). those where subjects had higher risk of colorectal cancer (CRC) as compared to the general population, for instance, individuals with a family history of CRC in first-degree relatives (n=7):**

1. Comstock S S, Hortos K, Kovan B, et al. Adipokines and obesity are associated with colorectal polyps in adult males: a cross-sectional study. *PloS one* 2014;**9**: e85939.
2. Hata K, Shinozaki M, Toyoshima O, et al. Impact of family history of gastric cancer on colorectal neoplasias in young Japanese. *Colorectal Disease* 2013; **15**:42-46.
3. Hong, Sung Noh, Tae Yoon Lee, and Sung-Cheol Yun. The risk of colorectal neoplasia in patients with gallbladder diseases. *Journal of Korean Medical Science* 2015;**30**:1288-1294.
4. Ng, Siew C., et al. Risk of advanced adenomas in siblings of individuals with advanced adenomas: a cross-sectional study. *Gastroenterology* 2016;**150**:608-616.
5. Suh, Sunghwan, et al. Korean type 2 diabetes patients have multiple adenomatous polyps compared to non-diabetic controls. *Journal of Korean Medical Science* 2011;**26**:1196-1200.
6. Touzin, Nadege T., et al. Prevalence of colonic adenomas in patients with nonalcoholic fatty liver disease. *Therapeutic Advances in Gastroenterology* 2011;**4**:169-176.
7. Yang, Xiao Bo, et al. Prevalence of colorectal neoplasm in Chinese patients with high‐risk coronary artery disease classified by the Asia–Pacific Colorectal Screening score. *Journal of Digestive Diseases* 2015;**16**:272-278.

### **(3). those that could not generate odds ratio for BMI category and colorectal adenoma (n=30)**

1. Corte, Crispin, et al. "Validation of the Asia Pacific Colorectal Screening (APCS) score in a Western population: An alternative screening tool." *Journal of gastroenterology and hepatology* 2016;**31**:370-375.
2. Betés, Maite, et al. Use of colonoscopy as a primary screening test for colorectal cancer in average risk people. *The American journal of gastroenterology* 2003;**98**: 2648-54.
3. Fischer, Roland, et al. Obesity and overweight associated with lower rates of colorectal cancer screening in Switzerland. *European journal of cancer prevention* 2013;**22**:425-430.
4. Giovannucci E , Colditz GA , Stampfer MJ et al. Physical activity, obesity, and risk of colorectal adenoma in women (United States) . *Cancer Causes Control* 1996;**7**:253-63 .
5. Gowda, Shilpa, et al. Low bone mineral density linked to colorectal adenomas: a cross-sectional study of a racially diverse population. *Journal of gastrointestinal oncology* 2015;**6**:165-71.
6. Huang H E, Yang Y C, Wu J S, et al. The relationship between different glycemic statuses and colon polyps in a Taiwanese population. *Journal of gastroenterology* 2014;**49**:1145-1151.
7. Lee, Go‐Eun, et al. Association between BMI and metabolic syndrome and adenomatous colonic polyps in Korean men. *Obesity* 2008;**16**:1434-1439.
8. Mannes, Gerd Alexander, et al. Relation between the frequency of colorectal adenoma and the serum cholesterol level. *New England Journal of Medicine* 1986;**315**:1634-1638.
9. Morois S, Mesrine S, Josset M, et al. Anthropometric factors in adulthood and risk of colorectal adenomas: The French E3N-EPIC prospective cohort. *American journal of epidemiology* 2010;**172**:1166-1180.
10. Nam S Y, Kim B C, Han K S, et al. Abdominal visceral adipose tissue predicts risk of colorectal adenoma in both sexes. *Clinical Gastroenterology and Hepatology* 2010;**8**:443-450.
11. Neugut AI, Lee WC, Garbowski GC, et al. Obesity and colorectal adenomatous polyps. *J Natl Cancer Inst* 1991;**83**:359–361.
12. Nock NL, Plummer SJ, Thompson CL, et al. FTO polymorphisms are associated with adult body mass index (BMI) and colorectal adenomas in African-Americans. *Carcinogenesis* 2011;**32**:748–756.
13. Oh, Tae‐Hoon, et al. Visceral obesity as a risk factor for colorectal neoplasm. *Journal of gastroenterology and hepatology* 2008;**23**: 411-417.
14. Olsen J, Kronborg O, Lynggaard J, et al. Dietary risk factors for cancer and adenomas of the large intestine. A case-control study within a screening trial in Denmark. *Eur J Cancer* 1994;**30**:53-60.
15. Otake, Sayaka, et al. Association of visceral fat accumulation and plasma adiponectin with colorectal adenoma: evidence for participation of insulin resistance. *Clinical Cancer Research* 2005;**11**:3642-3646.
16. Purdue MP , Mink PJ , Hartge P et al. Hormone replacement therapy, reproductive history, and colorectal adenomas: data from the Prostate, Lung, Colorectal and Ovarian (PLCO) Cancer Screening Trial (United States). *Cancer Causes Control* 2005;**16**:965-73 .
17. Pyo, Jeung Hui, et al. Is height a risk factor for colorectal adenoma? *The Korean journal of internal medicine* 2016;**31**:653-9.
18. Sato, Yumi, et al. Relation between obesity and adenomatous polyps of the large bowel. *Digestive Endoscopy* 2009;**21**:154-157.
19. Stadlmayr A, Aigner E, Steger B, et al. Nonalcoholic fatty liver disease: an independent risk factor for colorectal neoplasia. *J Intern Med* 2011;**270**:41–49.
20. Teetzmann A , Koch H , Oehler G . Coherence between body composition and benign colorectal neoplasia . *Med Welt* 2006;**57**:281-3.
21. Thoma, Matthew N., et al. Detection of colorectal neoplasia by colonoscopy in average-risk patients age 40–49 versus 50–59 years. *Digestive diseases and sciences* 2011;**56**:1503-1508.
22. Thompson C L, Berger N A, Chak A, et al. Racial differences in measures of obesity and risk of colon adenoma. *Obesity* 2012;**20**:673-677.
23. Trabulo, Daniel, et al. Metabolic syndrome and colorectal neoplasms: An ominous association. *World Journal of Gastroenterology* 2015;**21**:5320-7.
24. Tran F, Koo J H. Birthplace is not a determinant of colorectal adenomas. *World Journal of Gastroenterology* 2014;**20**:8606-11.
25. Terry MB, Neugut AI, Bostick RM, et al. Risk factors for advanced colorectal adenomas: a pooled analysis. Cancer Epidemiol Biomarkers.  *Prev* 2002;**11**:622–629.
26. Tsilidis KK, Brancati FL, Pollak MN, et al. Metabolic syndrome components and colorectal adenoma in the CLUE II cohort. *Cancer Causes Control* 2010;**21**:1–10.
27. Wolf L A, Terry P D, Potter J D, et al. Do factors related to endogenous and exogenous estrogens modify the relationship between obesity and risk of colorectal adenomas in women? *Cancer Epidemiology and Prevention Biomarkers* 2007;**16**:676-683.
28. Yamaji, Yutaka, et al. The effect of body weight reduction on the incidence of colorectal adenoma. *The American journal of gastroenterology* 2008;**103**:2061-7.
29. Yang W, Chang Y, Huang H, et al. Association between obesity, serum lipids, and colorectal polyps in old Chinese people. *Gastroenterology research and practice* 2013;**2013**;e931084
30. Zapatier, Jorge, et al. "Can adjusting BMI for age and sex provide for a better predictor of colonic neoplasia?." *European journal of gastroenterology & hepatology* 2015;**27**:974-980.

### **(4). those with symptomatic participants (n=7):**

1. Ashktorab, Hassan, et al. BMI and the risk of colorectal adenoma in African‐Americans. *Obesity* 2014;**22**:1387-1391.
2. Elwing JE, Gao F, Davidson NO, et al. Type 2 diabetes mellitus: the impact on colorectal adenoma risk in women. *Am J Gastroenterol* 2006;**101**:1866-1871.
3. Huang L, Wang X, Gong W, et al. The comparison of the clinical manifestations and risk factors of colorectal cancer and adenomas: results from a colonoscopy-based study in southern Chinese. *International journal of colorectal disease* 2010; **25**:1343-1351.
4. Nam JH , Yang CH . Clinical characteristics and risk factors of colon polyps in gyeongju and pohang area. *Korean J Gastroenterol* 2008;**52**:142-9.
5. Omata F, Brown W R, Tokuda Y, et al. Modifiable risk factors for colorectal neoplasms and hyperplastic polyps. *Internal medicine* 2009,**48**:123-128.
6. Sato Y, Nozaki R, Yamada K, et al. Relation between obesity and adenomatous polyps of the large bowel. *Digestive Endoscopy* 2009;**21**:154-157.
7. Kim B C, Shin A, Hong C W, et al. Association of colorectal adenoma with components of metabolic syndrome. *Cancer Causes & Control* 2012;**23**:727-735.

### **(5). those with BMI data obtained from self-reported questionnaires or no statement on the source of BMI data (n=20):**

1. Anderson, Joseph C., et al. Body mass index: a marker for significant colorectal neoplasia in a screening population. *Journal of clinical gastroenterology* 2007;**41**: 285-290.
2. Blanks, R. G., et al. Nationwide bowel cancer screening programme in England: cohort study of lifestyle factors affecting participation and outcomes in women. *British journal of cancer* 2015;**112**:1562-7.
3. Fu Z, Shrubsole M J, Smalley W E, et al. Lifestyle factors and their combined impact on the risk of colorectal polyps. *American journal of epidemiology* 2012;**176**:766-776.
4. Hassan, Cesare, et al. Impact of lifestyle factors on colorectal polyp detection in the screening setting. *Diseases of the Colon & Rectum* 2010;**53**:1328-1333.
5. Hermann, Silke, Sabine Rohrmann, and Jakob Linseisen. Lifestyle factors, obesity and the risk of colorectal adenomas in EPIC-Heidelberg. *Cancer Causes & Control* 2009;**20**:1397-1408.
6. Hong S N, Kim J H, Choe W H, et al. Prevalence and risk of colorectal neoplasms in asymptomatic, average-risk screenees 40 to 49 years of age. *Gastrointestinal endoscopy* 2010;**7**:480-489.
7. Jung, Yoon Suk, et al. Risk factors for colorectal neoplasia in persons aged 30 to 39 years and 40 to 49 years. *Gastrointestinal endoscopy* 2015;**81**:637-645.
8. Kahn, Henry S., et al. Risk Factors for Self‐Reported Colon Polyps. *Journal of general internal medicine* 1998;**13**:303-310.
9. Kaminski M F, Polkowski M, Kraszewska E, et al. A score to estimate the likelihood of detecting advanced colorectal neoplasia at colonoscopy. *Gut* 2014; **63**:1112-1119.
10. Kitahara C M, Berndt S I, de González A B, et al. Prospective investigation of body mass index, colorectal adenoma, and colorectal cancer in the prostate, lung, colorectal, and ovarian cancer screening trial. *Journal of clinical oncology* 2013;**31**:2450-2459.
11. Leitzmann, Michael F., et al. Adiposity in relation to colorectal adenomas and hyperplastic polyps in women. *Cancer Causes & Control* 2009;**20**:1497-1507.
12. Morimoto LM, Newcomb PA, Ulrich CM, et al. Risk factors for hyperplastic and adenomatous polyps: evidence for malignant potential? *Cancer Epidemiol Biomarkers Prev* 2002;**11**:1012–1018.
13. Nagata, Naoyoshi, et al. Visceral abdominal fat measured by computed tomography is associated with an increased risk of colorectal adenoma. *International journal of cancer* 2014;**135**:2273-2281.
14. Ruco, Arlinda, et al. Evaluation of a clinical risk index for advanced colorectal neoplasia among a North American population of screening age. *BMC gastroenterology* 2015;**15**:162-8.
15. Shaukat, Aasma, et al. Development and validation of a clinical score for predicting risk of adenoma at screening colonoscopy. *Cancer Epidemiology and Prevention Biomarkers* 2015;**24**:913-20
16. Steinmetz J, Spyckerelle Y, Gueguen R, et al. Alcohol, tobacco and colorectal adenomas and cancer. Case-control study in a population with positive fecal occult blood tests. *Presse medicale (Paris, France: 1983)* 2007;**36**:1174-1182.
17. Wernli KJ, Newcomb PA, Wang Y, et al. Body size, IGF and growth hormone polymorphisms, and colorectal adenomas and hyperplastic polyps. *Growth Horm IGF Res* 2010;**20**:305–309.
18. Wong M C S, Lam T Y T, Tsoi K K F, et al. A validated tool to predict colorectal neoplasia and inform screening choice for asymptomatic subjects. *Gut* 2014;**63**:1130-1136.
19. Yamamoto S, Nakagawa T, Matsushita Y, et al. Visceral fat area and markers of insulin resistance in relation to colorectal neoplasia. *Diabetes care* 2010;**33**:184-189.
20. Yamaji, Yutaka, Toru Mitsushima, and Kazuhiko Koike. Pulse-wave velocity, the ankle-brachial index, and the visceral fat area are highly associated with colorectal adenoma. *Digestive and Liver Disease* 2014;**46**:943-949.

### **(6). those with data on colorectal adenoma not derived from the whole colon and rectum (n=7)**

1. Betes M, Munoz-Navas MA, Duque JM, et al. Use of colonoscopy as a primary screening test for colorectal cancer in average risk people. *Am J Gastroenterol* 2003;**98**:2648–2654.
2. Bird CL, Frankl HD, Lee ER, et al. Obesity, weight gain, large weight changes, and adenomatous polyps of the left colon and rectum. *Am J Epidemiol* 1998;**147**:670–680.
3. Honjo, Satoshi, et al. The relation of smoking, alcohol use and obesity to risk of sigmoid colon and rectal adenomas. *Cancer Science* 1995;**86**:1019-1026.
4. Kim, Chang Sup, et al. The association of obesity and left colonic adenomatous polyps in Korean adult men. *Journal of preventive medicine and public health= Yebang Uihakhoe chi* 2005;**38**:415-419.
5. Larsen IK , Grotmol T , Almendingen K et al. Lifestyle as a predictor for colonic neoplasia in asymptomatic individuals. *BMC Gastroenterol* 2006;**6**:5-6.
6. Rabeneck, Linda, et al. Advanced proximal neoplasia of the colon in average-risk adults. *Gastrointestinal endoscopy* 2014;**80**:660-667.
7. Wong, Martin CS, et al. Identification of subjects at risk of proximal advanced neoplasia for colorectal cancer screening. *European Journal of Cancer* 2015;**51**:37-44.
